# Environmental Factors Influencing COVID-19 Incidence and Severity

**DOI:** 10.1146/annurev-publhealth-052120-101420

**Published:** 2022-01-04

**Authors:** Amanda K. Weaver, Jennifer R. Head, Carlos F. Gould, Elizabeth J. Carlton, Justin V. Remais

**Affiliations:** 1 Division of Environmental Health Sciences, School of Public Health, University of California, Berkeley, Berkeley, California, USA;; 2 Department of Epidemiology, School of Public Health, University of California, Berkeley, Berkeley, California, USA;; 3 Department of Environmental Health Sciences, Mailman School of Public Health, Columbia University, New York, NY, USA;; 4 Department of Earth System Science, Stanford University, Stanford, California, USA; 5 Department of Environmental and Occupational Health, Colorado School of Public Health, University of Colorado, Anschutz, Aurora, Colorado, USA;

**Keywords:** SARS-CoV-2, COVID-19, air pollution, chemicals, climate, built environment

## Abstract

Emerging evidence supports a link between environmental factors—including air pollution and chemical exposures, climate, and the built environment—and severe acute respiratory syndrome coronavirus 2 (SARS-CoV-2) transmission and coronavirus disease 2019 (COVID-19) susceptibility and severity. Climate, air pollution, and the built environment have long been recognized to influence viral respiratory infections, and studies have established similar associations with COVID-19 outcomes. More limited evidence links chemical exposures to COVID-19. Environmental factors were found to influence COVID-19 through four major interlinking mechanisms: increased risk of preexisting conditions associated with disease severity; immune system impairment; viral survival and transport; and behaviors that increase viral exposure. Both data and methodologic issues complicate the investigation of these relationships, including reliance on coarse COVID-19 surveillance data; gaps in mechanistic studies; and the predominance of ecological designs. We evaluate the strength of evidence for environment–COVID-19 relationships and discuss environmental actions that might simultaneously address the COVID-19 pandemic, environmental determinants of health, and health disparities.

## INTRODUCTION

The coronavirus disease 2019 (COVID-19) pandemic caused by the novel severe acute respiratory syndrome coronavirus 2 (SARS-CoV-2) has resulted in tens of millions of infections and millions of deaths worldwide (22). SARS-CoV-2 can be transmitted through the air via aerosols and droplets from talking, sneezing, and coughing (22, 136). Exposure—particularly prolonged and indoors—to the airspace of symptomatic and asymptomatic individuals is the dominant route of viral transmission, although transmission can also occur via direct physical contact and fomites (22, 54).

Although SARS-CoV-2 transmission has been documented in nearly all countries, the transmission dynamics and burden of morbidity and mortality have varied substantially across nations, regions, and even neighborhoods (17, 22, 145). This spatial and temporal heterogeneity is likely attributable to several factors, including nonpharmaceutical interventions, risk perception and human behavior, prevalence of comorbidities, structural determinants of health, and environmental conditions (52, 59, 76, 84, 92, 140, 146). Here, we evaluate the rapidly evolving COVID-19 literature—as well as research on related respiratory illnesses such as severe acute respiratory syndrome (SARS), Middle East respiratory syndrome (MERS), and influenza—to examine the effect of environmental conditions on SARS-CoV-2 transmission and COVID-19 incidence and severity.

**Figure 1:**
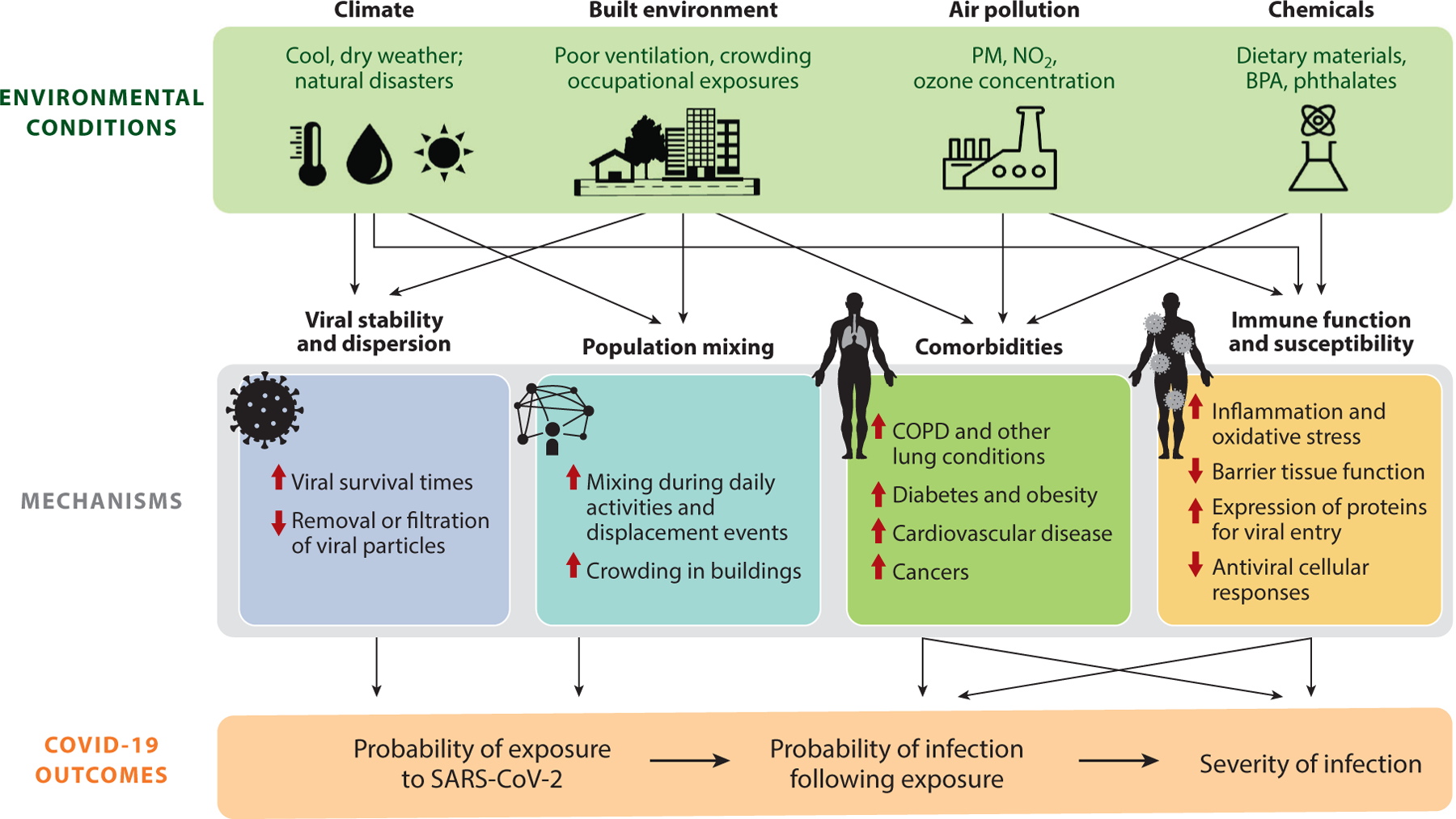
Schematic of the mechanisms through which environmental conditions influence SARS-CoV-2 exposure and COVID-19 susceptibility and severity. Thin arrows (*black*) represent pathways through which environmental conditions act on COVID-19. Bold arrows (*red*) indicate the direction of effect of the example exposures (*green text*) as either promoting (*upward arrow*) or suppressing (*downward arrow*) the associated mechanism. Abbreviations: BPA, bisphenol A; COPD, chronic obstructive pulmonary disease; COVID-19, coronavirus disease 2019; NO_2_, nitrogen dioxide; PM, particulate matter; SARS-CoV-2, severe acute respiratory syndrome coronavirus 2.

We propose that environmental factors influence SARS-CoV-2 via four main mechanisms (Figure 1): (*a*) exacerbating comorbidities and other respiratory conditions associated with severe COVID-19, (*b*) modifying host susceptibility to infection and/or disease severity through immune response modification, (*c*) regulating viral survival and aerosol transport, and (*d*) altering behavioral patterns that determine the frequency and intensity of pathogen exposure. We focus on the effect of four main factors—air pollution, chemical exposures, climate, and the built environment—on these pathways. In evaluating the strength of evidence for these mechanisms and environmental factors, we identify areas of uncertainty and emerging topics that could guide future research priorities (Supplemental Table 1). A critical evaluation of these relationships can strengthen estimates of the risk of COVID-19 attributable to environmental exposures and guide the design of interventions to slow virus spread, protect vulnerable populations from infection, and limit severe disease among those with elevated levels of risk.

## AIR POLLUTION EXPOSURES

Robust epidemiologic literature supports the role of short- and long-term air pollution exposure in elevating the risk of respiratory viral infections and impairing immune function, raising hypotheses regarding its potential effects on COVID-19 incidence, severity, and disparities. Emerging evidence suggests that air pollution may elevate the risk of infection and mortality from COVID-19 via two key pathways (Figure 1): (*a*) modifying host susceptibility to infection and disease severity, and (*b*) elevating the risk of comorbidities. The former may be mediated by upregulation of proteins critical to viral entry and by immune system suppression from oxidative stress, epithelial damage, and pulmonary inflammation.

### Protein Expression and Immune Impairment

Exposure to particulate matter (PM) can increase the expression of angiotensin-converting enzyme 2 (ACE2) and transmembrane protease serine type 2 (TMPRSS2), proteins critical to SARS-CoV-2 entry into host cells (56). Upregulation of proteins necessary for viral entry may lead to higher viral load, thereby elevating the risk of severe COVID-19. Laboratory studies show that these proteins are upregulated in response to short-term smoke and PM exposure (115, 144). Studies in mice have documented a dose-dependent upregulation of ACE2 and TMPRSS2 proteins following a 4-24-h exposure to smoke (144) and, separately, an intratracheal exposure to a 50-*µ*g PM solution (115). Upregulation occurred more in alveolar type 2 (AT2) cells—potential targets of SARS-CoV-2 (38)—and macrophages (115).

Immunological impairment prior to COVID-19 infection, induced by long-term exposure to PM, NO_2_, and ozone, may also increase the risk of COVID-19 infection and/or its severity. Pulmonary barrier tissues and mucociliary clearance processes form the first line of defense against invading pathogens (42, 57). Exposure to air pollution renders these defenses less effective at preventing host pathogen invasion when damaged by particulate invasion (44). Oxidative stress associated with air pollution exposure (e.g., to NO_2_, ozone, or PM) can also yield barrier tissue and respiratory system impairment (27, 72). In addition, NO_2_ exposure can lead to inflammation and impair tissue defenses and phagocytic activity by depleting the antioxidant pool (86), while ozone exposure can trigger an inflammatory response and systematic oxidative stress (131). Macrophages produce antigens to clear pathogens; however, evidence has shown that alveolar macrophages exposed to PM_10_ produced 50% less viral antigen in response to respiratory syncytial virus (RSV), increasing the risk of infection (10). Severe COVID-19 is associated with high inflammation and elevated levels of inflammatory cytokines (89). Once pathogens establish themselves, inflammation of pulmonary tract mucus membranes resulting from air pollution exposure may contribute to a higher risk of severe COVID-19 outcomes through compounded inflammation (86). Both inflammation and oxidative stress are associated with aging (46, 47, 79), and their upregulation by PM, NO_2_, and ozone exposure may contribute to even greater age-related COVID-19 mortality (86).

### Elevated Comorbidity Risk

PM exposure has been associated with an increased risk of chronic obstructive pulmonary disease (COPD), diabetes, and hypertension (73, 138), conditions associated with an increased risk of intensive care unit (ICU) admission, ventilation, and death from COVID-19 by harming the respiratory system and/or increasing inflammation (52). In addition, PM_2.5_ exposure is linked to atherosclerosis (71), cardiovascular disease, congestive heart failure, arrhythmias, lung cancer (73, 138), and community-acquired pneumonia in older adults (99). Meanwhile, ozone exposure has been associated with hypertension, arrhythmias, and cardiovascular-related hospital admissions, among other cardiovascular conditions (131, 138). Short-term NO_2_ exposure has been associated with respiratory and cardiac mortality, including conditions such as ischemic heart disease and heart failure (26). Short-term exposures to PM_10_, ozone, and NO_2_ have also been linked to heart failure, asthma, pneumonia, and influenza hospital admissions (138). Such preexisting comorbidities elevate the upstream risk of poor COVID-19 outcomes (52) due to poorer baseline health.

### Epidemiologic Studies

Complementary to evidence of these plausible mechanisms, epidemiologic evidence shows a robust association between air pollution and the incidence of a number of respiratory viral infections, including SARS, influenza, and RSV (40). Several ecological studies in the United States, China, Italy, England, and the Netherlands have found evidence that areas with poorer air quality are more likely to have elevated COVID-19 incidence and mortality (28, 29, 43, 134, 140, 150). For example, in a study examining the relationship between long-term (from 2000 to 2016) average PM_2.5_ concentrations and COVID-19 mortality rate in 3,089 US counties, adjusted for 20 county-level confounders, researchers found that a 1 *µ*g per m^3^ increase in PM_2.5_ was associated with an 11% [95% confidence interval (CI): 6–17%] increase in county-level COVID-19 mortality rate (140). Using individual-level data, a cohort study of US veterans found that a 1.9 *µ*g per m^3^ increase in PM_2.5_ concentration was associated with a 10% (95% CI: 8–12%) increase in risk of COVID-19 hospitalization, among individuals with COVID-19 (15). Short- and medium-term air pollution exposures, such as those experienced during wildfire events, may also influence COVID-19 outcomes (149). A time-series analysis of PM_2.5_ exposure in wildfire-affected counties in California found that a daily median increase of 10 *µ*g per m^3^ over 28 days was associated with a 12% (95% CI: 8–16%) increase in COVID-19 cases and an 8% (95% CI: 2–15%) increase in COVID-19 deaths (149).

Independent of PM_2.5_, emerging evidence supports a positive association between COVID-19 and NO_x_ (24, 81, 134) and mixed directionality of association with ozone exposure (134, 150). For example, long-term exposure to NO_2_ has been associated with an elevated risk of COVID-19 cases and mortality across neighborhoods in Los Angeles (81). Researchers found that an 8.7-ppb increase in mean annual NO_2_ concentration was associated with a 16–31% increase in the COVID-19 case rate and a 35–60% increase in mortality rates across model specifications (81). More recently, a multiethnic cohort study from Kaiser Permanente Southern California found that one year of exposure to near-roadway nonfreeway NO_x_ was significantly associated with an 8% increase in odds of COVID-19-related ICU admission (95% CI: 2–16%) and an 8% increase in hazard of death (95% CI: 1–16%), adjusting for sociodemographic covariates and regional air pollutants (PM_2.5_ and NO_2_) (24). The extent to which ozone exposure influences COVID-19 is less clear, as the evidence base is limited and studies have found mixed directionality of association (134, 150).

Air pollution exposure disproportionately impacts racial and ethnic minorities and those of low socioeconomic status in both the outdoor (11, 53) and indoor environments (2), potentially contributing to unequal COVID-19 incidence and mortality rates observed across racial, ethnic, and other groups in the United States and globally (9, 97). For example, a cross-sectional study found that Black and Hispanic populations experienced age-standardized COVID-19 mortality rate ratios that were 3.6 and 2.2 times higher, respectively, than that of non-Hispanic White populations in the United States (9). Furthermore, Bowe et al. (15) observed that race and neighborhood modify the effect of air pollution on COVID-19 outcomes, with an elevated risk for Black populations ( *p* = 0.045) and those living in low-socioeconomic-status neighborhoods ( *p <* 0.001).

### Critical Gaps

Epidemiologic studies assessing the relationship between air pollution and COVID-19 incidence to date have been subject to methodologic limitations that may introduce bias and limit causal inference, as discussed elsewhere (12, 135). First, most studies to date have employed ecological designs, associating group-level air pollution exposures with aggregate COVID-19 outcomes over a broad geographic domain (e.g., 28, 29, 43, 134, 140), thus preventing inference at the individual level. Such designs may be subject to bias from residual confounding, as individual-level confounders (e.g., race, age, sex, smoking status) are also aggregated. Important group-level confounders such as population density, testing rate, and pandemic stage have not always been accounted for in the current literature (43, 135). Second, many air pollution epidemiologic studies rely on COVID-19 disease incidence estimated from surveillance data, resulting in an outcome inherently conditioned on cases that sought care and obtained testing, thus introducing selection bias if other factors that induce an association between air pollution and testing rates are not accounted for in the analysis. Third, ambient air pollution exposure estimates are subject to misclassification from a variety of factors (e.g., inadequate ground monitoring, limitations of remotely sensed data, failure to capture individual heterogeneity in exposure) that add additional uncertainty to analyses. Finally, many of the studies discussed in this review focus on the relationship between long-term exposures to air pollution and COVID-19 incidence and severity. Yet, short-term exposures to high concentrations of pollutants, such as might be experienced during a wildfire event, may also play a role in exacerbating COVID-19 morbidity and mortality (149).

Studies of the relationship between air pollution and COVID-19 incidence will benefit from personal monitoring to estimate individual-level air pollution exposures indoors, outdoors, and across all activities (e.g., working, driving, walking). While personal monitoring is expected to benefit the air pollution epidemiology community at large, it would also advance our understanding of key exposures relevant to COVID-19, such as short-term changes in filtration systems, mobility, and shifts in emissions associated with changing commute patterns. Furthermore, monitoring air pollution exposures at the individual level may expose economic and racial disparities in access to filtration systems that remove both air pollutants and aerosolized viral particles. Prospective cohort studies based on personal measurements can reduce exposure misclassification while permitting confounder control at the individual level. However, these approaches are resource intensive; in their absence, case-crossover studies may be a feasible study design for examining the effect of group-level exposures on individual outcomes while controlling for time-invariant, individual-level confounders.

## CHEMICAL EXPOSURES

Exposures to a wide variety of chemicals, including metals (e.g., arsenic, cadmium, and lead) and endocrine-disrupting chemicals [EDCs; e.g., bisphenol A (BPA), phthalates, and perfluorinated chemicals (PFCs)], may be risk factors for COVID-19 susceptibility and severity (40, 127). Although direct evidence supporting these hypotheses currently remains limited, we outline potential mechanisms for and available evidence of how chemical exposures may lead to increased susceptibility to COVID-19 infection and, given infection, increased severity of COVID-19. We focus on two key pathways (Figure 1): (*a*) modifying host susceptibility to infection and disease severity, and (*b*) elevating the risk of comorbidities. We highlight how chemical exposures may contribute to immune impairment through barrier organ dysfunction, inflammation, and oxidative stress, as well as elevate the risk of both respiratory and nonrespiratory diseases associated with severe COVID-19.

### Immune Impairment

Like air pollution exposures, chemical insults have damaging effects on barrier organ function, thereby increasing the likelihood of viral entry into the host and increasing the likelihood of COVID-19 infection (103). For example, chemical exposures can damage lung epithelial cells (55) and interfere with the tight junctions between epithelial cells (20), yielding reduced protection against viral infection over time through a more permeable airway and pulmonary epithelium. Chemical exposures—e.g., to arsenic (110) and cadmium (143)—can also lead to reduced mucociliary clearance, increasing pathogen time within the host and viral infection risk. Furthermore, chemical exposures—e.g., PFCs (132), arsenic (6), cadmium (69), and lead (39)—can weaken immune function and reduce resistance to infection through a variety of mechanisms, including (*a*) altered T cell proliferation and activation by reduced interleukin-2 production, an important cytokine in cell-mediated immune function; (*b*) altered T cell structure; (*c*) altered B cell maturation; and (*d*) direct cytotoxicity to monocytes, lymphocytes, and macrophages. After infection, chemical exposures, including cadmium, arsenic, phthalates, and BPA, may also increase the risk of severe COVID-19 through aberrant or exaggerated immune responses marked by oxidative stress, inflammation, immune dysfunction, and cell death (60, 103, 110). Such exaggerated immune responses are associated with multiple organ system failure, COVID-19 hospitalization, and death (89).

Complementary to evidence of these plausible mechanisms through which chemical exposures may increase the risk of viral infection and severe disease, epidemiologic studies have also identified positive associations between chemical exposures and viral infections, including arsenic exposure and the risk of hepatitis A, B, and E infections (6) as well as lower respiratory infections (6); cadmium exposure and the risk of mortality from influenza and pneumonia (102); exposure to BPA and phthalates and the risk of respiratory tract infections (49); and exposure to polychlorinated biphenyls and the risk of acute respiratory infections (35).

### Elevated Comorbidity Risk

Because a history of respiratory dysfunction can lead to elevated risk of severe COVID-19, long-term exposure to metals [e.g., arsenic (118), cadmium (48), and lead (14)] that are associated with lung function impairment, respiratory symptoms, and respiratory diseases—e.g., COPD and interstitial lung disease—may elevate upstream risk of severe disease after viral infection (52). Mechanisms have been proposed on the basis of both epidemiologic evidence and animal studies. For example, arsenic, cadmium, and lead may permanently alter lung structure and function via extensive tissue inflammation, altered expression of structurally important extracellular matrix genes, and impaired repair mechanisms in the lung epithelium (48, 118). These physical changes to the airways then lead to restrictive and/or obstructive impairments to the lung. Additional emerging evidence indicates that exposures to pesticides, phthalates, and PFCs and per- and polyfluoroalkyl substances (PFAS) may be associated with impaired lung function (65, 106, 111). However, the exact mechanisms through which these chemical exposures may affect lung function are unknown, though some researchers have suggested that exposures act through oxidative stress, which is associated with declining lung function and COPD (66).

Epidemiologic, clinical, and mechanistic evidence suggests several links between chemical exposures and comorbidity risk factors for COVID-19 severity beyond respiratory dysfunction, including hypertension (52), obesity (119), diabetes (98), and cancer (64). For instance, metals—particularly mercury, lead, cadmium, and arsenic—are associated with cardiovascular disease of atherosclerotic origin (130). Cadmium and lead exposure have well-established associations with hypertension, as well as with atherosclerosis from increased aortic atherosclerotic plaque burden (130). Epidemiologic studies have shown links between obesity and type 2 diabetes and various metals (e.g., mercury, cadmium, lead, and arsenic) and EDCs (e.g., BPA, phthalates) (8). Obesity is characterized by constant chronic inflammation, causing a delayed and inferior immune response. As reviewed elsewhere (98, 119), obesity and type 2 diabetes are risk factors for poor COVID-19 prognosis. A number of metals (e.g., arsenic, cadmium) and chemicals (e.g., polycyclic aromatic hydrocarbons) are considered carcinogens (64, 103) and may be important environmental correlates of COVID-19, given that individuals with cancer are considered high risk for infection and severe disease.

### Evidence

At this time, there remains only limited direct evidence of the potential relationship between chemical exposures and COVID-19. One study used a computational systems biology approach to characterize pathways through which EDCs (notably perfluorooctanoic acid and perfluorooctane sulfonic acid) may lead to increased predisposition to severe COVID-19. The investigators identified IL-17 and advanced glycation end products and the associated receptor signaling pathways as important potential avenues, given their association with stress and inflammation (139).

Several studies have examined the concentrations of metals/metalloids and toxic chemicals in COVID-19 patients (51, 147, 148). One study found elevated levels of urinary chromium, cadmium, mercury, and lead in patients with worse outcomes (severe versus nonsevere; deceased versus recovered) (148), while a similar study by the same group found higher levels of whole blood chromium and cadmium concentrations—but also lower arsenic concentrations—independent of sex, comorbidities, and metal concentrations (147). Further research leveraged Danish biobanks to obtain plasma samples from 323 subjects with known SARS-CoV-2 infection to show that, among five PFAS measured, perfluorobutanoic acid was associated with increased severe COVID-19, even when adjusted for sex, age, comorbidities, and sample batch (51).

### Critical Gaps

Studying the effect of chemical exposures on COVID-19 incidence and severity comes with several challenges. First, populations are widely exposed to many chemicals and stressors from a variety of sources; thus, identifying the impacts of a single chemical or stressor is a challenge and is also subject to interactions with other chemical exposures. Second, chemical exposures vary over the life course, and the impacts of the timing of exposure are often uncertain. In some cases, prenatal and early-life exposures during critical windows of immune development can lead to immune function impairments and increased risk of infections later in life (19). In addition, growing evidence indicates both that some exposures lead to epigenetic changes that can affect later generations and that exposures in utero can affect the development of the immune system, altering immune function later in life.

Direct evidence demonstrating that chemical exposures affect COVID-19 risk and severity is still lacking. Even for long-studied chemicals and metals, including lead, arsenic, cadmium, bisphenols, phthalates, and PFAS, the precise impacts and mechanisms through which they act on the human body are still subject to significant uncertainty. Still, taken together, it is clear that multiple metal and chemical exposures may impact host susceptibility to COVID-19 infection and severity of COVID-19 given infection, particularly through their associations with health conditions that predispose individuals to severe COVID-19.

## CLIMATIC CONDITIONS

Many infectious respiratory diseases, including influenza and those caused by other coronaviruses, exhibit seasonal patterns that are partially explained by climatic conditions affecting virus survival, seasonal immunity, and population mixing. A growing body of epidemiologic evidence suggests that SARS-CoV-2 transmission risk is higher at lower ambient temperatures and at lower humidity (e.g., 82, 84, 88, 100, 122, 129). We focus on climate conditions implicated as potential drivers of SARS-CoV-2 infection risk and COVID-19 susceptibility and severity—temperature, humidity, UV radiation, and extreme weather events—and detail potential mechanisms (Figure 1) by which these factors may influence viral persistence in the environment, immune system function, and population movement and human behaviors.

### Virus Survival

The relationship between cooler temperatures and lower humidity and increased risk of COVID-19 (82, 84, 100, 122, 129, 141) may be explained by the effects of these conditions on viral persistence in the environment. Temperature and relative humidity (RH) can modulate the decay rate of viruses within aerosols (80) as well as droplet size through evaporation (85). Laboratory studies have shown that SARS-CoV-2 exhibits greater stability at lower temperatures (25, 87, 95, 108, 113). For example, when contained within liquid human nasal mucus and sputum, the half-life of the virus consistently declined with increasing temperatures (4°C, 21°C, and 27°C) (87). Using a mechanistic model to predict the impact of temperature and RH on SARS-CoV-2 stability (95), researchers found that virus survival was highest at low temperatures across all humidity levels considered. The sensitivity of virus to temperature is strongest in the absence of UV light (108). In dark conditions, the half-life of SARS-CoV-2 on simulated human secretions fell from a few days at 20°C to a few hours at 40°C (113), and at 20°C, 10 times more active virus remained on surfaces 7 hours after inoculation compared with 35°C (108).

The relationship between humidity and SARS-CoV-2 survival is more nuanced, with some evidence suggesting that the relationship is convex, such that stability is highest under both high and low RH (95). While higher RH may slow the evaporation of respiratory droplets (85), some controlled studies of human nasal mucus and sputum (87) and viral aerosols (33) show that SARS-CoV-2 decays more rapidly at higher RH. The shared conclusion that virus survival is greater in relatively dry conditions is consistent with evidence for influenza, where transmission is optimal at a low absolute humidity (124).

UV radiation also appears to reduce viral stability, consistent with previous evidence that single-stranded RNA viruses such as SARS-CoV-2 are generally susceptible to inactivation via UV radiation (108, 116, 120). Studies of SARS-CoV-2 in laboratory settings have shown rapid viral decay under simulated sunlight (33, 112, 116, 120). For example, one laboratory-based simulation found that 19 min of exposure to simulated winter and fall UV conditions inactivated 90% of SARS-CoV-2, a degree of inactivation achieved in just 8 min of simulated summer conditions (120). This finding is similar to other studies that estimated inactivation of 90% of SARS-CoV-2 after 11–34 min (116) and 14.3 min (112) of midday sunlight exposure in North America. While UV radiation appears to be the driving factor for viral stability in sunlight-exposed areas (108), most SARS-CoV-2 transmission occurs indoors (105), where the role of sunlight in regulating transmission may be limited.

### Immune System Effects

Both the adaptive and innate immune responses have been shown to be modulated by seasonal fluctuations. In particular, the cold, dry conditions of the winter months can suppress the immune system through a number of mechanisms, including reduced mucociliary clearance (83) and reduced levels of vitamin D due to reduced sun (UVB) exposure (18). Cellular immune response may also be affected by temperature and humidity. For example, mouse airway epithelial cells initiated a more robust antiviral response at warmer temperatures as compared with cooler temperatures (45), and mice exposed to low-humidity conditions were more susceptible to influenza (70). However, the effects of seasonal fluctuations in immune response on COVID-19 susceptibility and severity are still largely unknown.

### Population Mixing

Extreme weather events can alter transmission by affecting population mixing. On the one hand, weather-related closures of schools or businesses can weaken social connections and reduce disease incidence. Such was the case in Seattle, when a snowstorm during the height of influenza season forced workplace and school closures, leading to a reduction of 16–95% in contact rates and a reduction of 3–9% in seasonal influenza incidence (61). On the other hand, extreme weather events such as hurricanes, wildfires, and earthquakes can also displace populations, forcing individuals to aggregate in shelters and leading to elevated population mixing and infection spread (30). Nominal weather patterns may also play a limited role. For instance, early in the pandemic, researchers found that people in the United States were more likely to go to parks in warmer weather, but no association was found between temperature and encounter rate (142). However, populations may also gather in closer contact indoors in the colder months, settings known to be dominant loci of transmission. In an analysis of 30 counties, meteorological factors were found to have a marginal direct effect on COVID-19 cases and deaths, reflecting action on virus stability, but meteorological factors demonstrated significant indirect effects via human mobility (36).

### Epidemiologic Studies

Early time-series analyses found consistent evidence that COVID-19 incidence and mortality were negatively associated with ambient temperature and humidity in temperate and tropical regions (82, 141). A systematic review of 17 studies of temperature, humidity, and SARS-CoV-2 that were published before March 24, 2020, found consistent evidence that SARS-CoV-2 transmission was associated with low temperatures and low humidity (88). Since then, studies have generally shown similar, albeit more nuanced, results. For example, a study of reported COVID-19 cases in 54 English cities observed negative nonlinear associations between temperature and reported cases; cooler, drier conditions were associated with the greatest risk of incidence (100). A study of 26 countries found modest, nonlinear association between mean temperature and *R*_*e*_ that peaked at 10.2°C; a weak nonlinear association with RH; and no association with solar radiation, wind speed, and precipitation (122). An investigation of the nonlinear relationships between *R*_e_ and meteorological factors in the United States found that *R*_e_ peaked between 10°C and 20°C and increased at lower levels of specific humidity and solar radiation (84). Another global study found that a one standard deviation increase in local UV was associated with 0.97% decrease in COVID-19 growth rate over the subsequent 2.5 weeks (21).

While meteorological conditions may facilitate or limit transmission, mitigation policies (e.g., public health measures) and behaviors are likely to play a larger role in determining the degree of transmission (114, 122). Interventions adopted by governments (e.g., masking, distancing, policies as measured by the government response index) were found to explain five times as much variation in *R*_e_ as mean temperature in the early stages of local epidemics (122). Another global study similarly found that weather and demography explained only 17% of the variation in maximum COVID-19 growth rates, while country-specific effects explained an additional 19% (92). One study of COVID-19 in the United States prior to large-scale vaccination efforts found the fraction of *R*_*e*_ attributable to meteorological factors to be 17.5%, with effects proportionally attributed to temperature (3.7%), humidity (9.4%), and UV radiation (4.4%) (84). Another study from this same setting and time period found that *R*_e_ increased as temperatures cooled but that the influence of population density on *R*_e_ was 1.4 times greater than the influence of temperature (129).

Furthermore, in the phase of the pandemic preceding large-scale global implementation of vaccinations (i.e., late 2019 through early 2021), the majority of the global population was susceptible to infection, and the effects of climate as a driver of spread are likely to be minimal as compared with the effects of contact rates and public health measures. One study that focused on the beginning of the pandemic through the summer of 2020 estimated that high supply of susceptible individuals strongly limits the role of climate, suggesting that climate may become more important in the longer term, as populations become immunized through vaccination and prior infection (7).

### Critical Gaps

Many of the challenges and limitations discussed previously—such as for COVID-19 air pollution epidemiology—arise when examining the relationship between the climate and COVID-19. These include methodological challenges, including unmeasured confounders, limitations of surveillance data, aggregation of exposures across broad regions, and a lack of indoor environmental monitoring, as well as a lack of mechanistic understanding of the effect of climate on SARS-CoV-2 immune response. In addition, the range of environmental conditions used in laboratory studies of virus survival are not always representative of real-world environmental conditions (e.g., conditioned indoor spaces). For example, few laboratory studies have considered RH below 30%, even though in winter months and in arid climates indoor RH may frequently be below 10%. Moreover, while it is commonly understood that the climate influences human behavior in ways that may impact SARS-CoV-2 transmission, uncertainty remains regarding the role of human movement and contact patterns, especially as government intervention has been shown to explain significant variation in transmission in the early stages of the pandemic (122).

## BUILT ENVIRONMENT

Features of the built environment moderate the spread of infectious agents through regulation of indoor air quality and ventilation in residential and occupational settings, maintenance of ambient temperature, determination of crowding, and the distribution of health resources and hazards within neighborhoods (Figure 1) (104). The built environment has played a key role in the transmission of other novel viral respiratory infections, including the 2003 outbreak of SARS, where transmission was facilitated by unsealed floor drains and a poor ventilation system in the Amoy Gardens apartment complex (104). Here, we focus on both the interior characteristics of buildings and the structure and design of neighborhoods as key environmental determinants of COVID-19 incidence and severity. We discuss, but do not comprehensively review, the influence of the built environment on occupational exposures, reserving the topic for future research and synthesis.

### The Indoor Environment

People in the United States and other settings spend approximately 90% of their time indoors (74), and SARS-CoV-2 is acquired predominately by transmission in the indoor environment (105). Indoor transmission can be regulated by ventilation, filtration, and climate control, which in part determine the density and survival of pathogens and thus infection risk (76, 78). While sanitation can reduce pathogen density on surfaces, risk of infection from touching a contaminated surface has been estimated to be low for COVID-19 (*<*4 in 10,000 surface touches) (54). Given the minimal role of fomite transmission in the COVID-19 pandemic, the importance of surface sanitation in indoor environments is expected to be minimal compared with ventilation and filtration.

Ventilation that achieves 4–6 air changes per hour (ACH) is thought to reduce airborne concentrations and mitigate airborne spread of SARS-CoV-2 (4). However, few nonspecialized buildings are designed to mitigate airborne transmission (93), and many indoor settings do not achieve infection-risk-based ventilation targets. For instance, more than half of California elementary school classrooms did not meet state standards for classroom ventilation (2.8 ACH) in a 2013 assessment (91). Optimal ventilation rates are challenging to define for transmission prevention because they vary on the basis of individual risk, occupancy, and activity (93). Ventilation alone is not sufficient in many scenarios, necessitating additional filtration practices. Filtration with minimum efficiency rating value (MERV) 13 or high-efficiency particulate air (HEPA) filters can reduce viral particle concentrations, with MERV 13 filters capturing 66% of 0.3–1.0 *µ*m particles and HEPA filters exhibiting near 100% capture efficiency (4).

Many buildings are maintained at temperature and humidity conditions within a comfortable range, which could protect SARS-CoV-2 from destabilizing extremes (95). SARS-CoV-2, like other human coronaviruses (94, 95), can survive in typical climate-controlled conditions of moderate temperature and low humidity. Homes, businesses, and other buildings that are equipped with climate control may inadvertently maintain suitable transmission environments. At the same time, lower-income and minority populations are more likely to live in buildings without climate controls such as air conditioning (101), which can pose additional health risks from prolonged exposures to extreme temperatures and mold (59, 101, 126). Furthermore, the role of temperature is complicated by behavioral responses to indoor conditions. For instance, in a high school in Israel, temperatures exceeding 40°C may have been less suitable for SARS-CoV-2 survival but prompted students and teachers to remove their masks, leading to an outbreak of COVID-19 cases (31).

The physical structure of homes, businesses, and other buildings can also facilitate or impede the ability to social distance and/or quarantine. Crowding can raise SARS-CoV-2 transmission risk by increasing interpersonal contact frequency and duration. Indeed, SARS-CoV-2 transmission was associated with large household size, crowding, and socioeconomic status among pregnant women in New York City (41), and high SARS-CoV-2 seroprevalence (22.1%) was observed among agricultural workers, many living in overcrowded housing (77). In addition, US counties with a high percentage of households with poor housing, defined as overcrowded, overpriced, and/or missing kitchen and plumbing facilities, also had a higher incidence of—and mortality from—COVID-19 (3). Finally, crowding in carceral facilities has resulted in several large COVID-19 outbreaks, and the case rate among prisoners has been estimated at 5.5 times that of the general population (117). Increased crowding in Massachusetts prisons (defined as a 10% increase in occupancy over design capacity) was also associated with a 14% increase in COVID-19 incidence (95% CI: 3–27%) (75).

### Multiscale Structural Factors

At the neighborhood scale, the built environment codifies structural inequalities through how people live, work, learn, and socialize, creating a highly racialized, heterogeneous geography of risk (145). These structural factors create environments where historically marginalized groups are more likely to be exposed to pathogens, become infected, and die from infection (104). Historical practices, including redlining and the diversion of public resources away from minority communities, have decreased access to quality education, health care, work, housing, and food (1, 32, 59, 107, 145). Thus, neighborhoods can be chronically detrimental to community health and particularly risky for infectious disease transmission.

Racial and socioeconomic segregation may elevate the risk of COVID-19 transmission by creating barriers that separate communities from essential resources, elevate exposure to SARS-CoV-2, and concentrate transmission in segregated communities (1, 58, 146). In the United States, ongoing racial and socioeconomic segregation groups people in neighborhoods with fewer essential services and complete streets investments, including grocery stores, bus and bike infrastructure, green spaces, and quality housing (16, 59, 137). This historic disinvestment contributes to higher burdens of both infectious disease and noncommunicable comorbidities in majority-minority, low-income neighborhoods, as compared with majority White, wealthy neighborhoods (5), through reduced access to nutritious foods, limited active and safe travel, and increased household exposures to pathogens and temperature extremes (59, 126, 137). For example, US counties that were one standard deviation above the mean of residential segregation (as measured by the multigroup relative diversity index) experienced COVID-19 mortality and infection rates that were 8% (95% CI: 2–14%) and 5% (95% CI: 1–10%) higher than the mean when accounting for 50 demographic, density, social capital, health risk, health system capacity, air pollution, essential business, and political view variables (133). In Kolkata, India, COVID-19 spatial clusters were found to have high correspondence with low-income areas with high values on the index of multiple deprivation (a measure of housing conditions and amenities, assets, water, sanitation, hygiene access, and gender disparities in literacy and work) (37).

Health system access is also lower in high-minority and lower-income areas, with medical infrastructure, insurance coverage, and other health resources distributed highly inequitably (137). The persistence of large populations who are uninsured or underinsured has been a long-standing source of health inequity, resulting in disparities in COVID-19 outcomes among racial minorities and the poor (9, 145, 146). For instance, minorities encountered longer travel times and thus reduced access to SARS-CoV-2 testing sites in the United States (107). Accordingly, neighborhoods in Chicago with lower health insurance coverage were associated with higher COVID-19 mortality (17).

Low-income and minority populations are also more likely to work in essential services, are unable to work remotely and social distance, and thus experience elevated levels of exposure (32, 58, 62). As such, New York City neighborhoods where daily movement and population outflow were unchanged by social distancing policies had the highest exposure density and were composed of a higher percentage of racial minorities and health care support workers as compared with neighborhoods where movement was limited by social distancing policies (58). In addition, these neighborhoods had lower income and educational attainment, as well as higher unemployment rates and large household size (58).

### Critical Gaps

Key uncertainties limit our understanding of the relationship between the built environment and COVID-19, including major challenges in resolving the built environment exposome and its causal linkages to health outcomes (34). People vary greatly in their daily routines, traveling from home, work, school, and public and commercial spaces, and may exhibit location-dependent and highly personal exposures to pathogens, chemicals, and other stressors (63). Given that many studies to date leverage neighborhood-level data, exposure misclassification may limit accurate inference on the role of the built environment. Significant research efforts are needed to clarify causal relationships between the exposome, structural characteristics, and health outcomes (34). Such work will be critical to informing future building codes, city planning agendas, regulation of environmental hazards, and policy reforms that address housing and other built environment disparities.

## EMERGING DIRECTIONS AND FUTURE RESEARCH

Key emerging areas of inquiry and investigation are set to expand our understanding of the environmental dimensions of the COVID-19 pandemic. While environmental exposures may influence SARS-CoV-2 transmission, the pandemic has also impacted exposures. For instance, lockdown policies may lead to greater concentrations of residential indoor air pollution (96) and reductions in ambient concentrations (13). Global economic shocks have likely increased energy insecurity, leading some to engage in risky behaviors to meet household energy needs, including the use of highly polluting biomass fuels for cooking or gas ovens for heating, and forego other basic needs (90). Shifting time activity from workplaces, schools, and commercial spaces to residential environments has reignited interest in a fuller accounting of environmental exposures across indoor and outdoor environments. The growing availability of mobile device data indicates profound shifts in telework, shopping from home, and public transit usage (121), highlighting the need to reassess assumptions about time activity. These changes have yielded uncertain impacts on environmental exposures and will likely be the subject of research for years to come.

Wastewater-based surveillance and epidemiology—to reveal areas of persistent infection and targets for vaccination efforts, for instance—are additional areas that will be potentially critical to long-term prevention and recovery efforts (128). Wastewater surveillance systems have been established across the United States to monitor COVID-19 infection extent and variants in communities. Given the long-term risk of ongoing transmission in low- and middle-income countries, and the limited capacity of clinical surveillance networks, establishment of wastewater surveillance has particular public health potential in these settings. Though such approaches have been used to monitor for outbreaks of other diseases (e.g., poliomyelitis), further research will be needed to accommodate the scale of the COVID-19 pandemic, the threat of emerging variants, variable sewer quality and access, and challenges defining sewersheds.

As vaccination coverage increases, climate may play a larger role in determining COVID-19 infection in comparison to contact dynamics, potentially leading to seasonal transmission patterns (7). Other human coronaviruses and influenza exhibit pronounced seasonality that peaks in the winter months, driven by the cyclical nature of temperature, humidity, behavior, and waning immunity (94). Laboratory experiments have shown that SARS-CoV-2 is similarly environmentally sensitive (25, 87, 95, 108, 113). If SARS-CoV-2 immunity wanes at rates similar to those of other coronaviruses, one study predicts recurrent winter outbreaks (68). Future research should address interactions between climate and immunity—including cross-immunity, seasonal cycles, and heterogeneity in the duration of immunity from multiple vaccines and previous infections—to inform the likelihood of seasonal circulation of SARS-CoV-2.

The COVID-19 pandemic has emerged as climate change continues to influence temperature and precipitation patterns, induce extreme weather events, and force population displacement, leading to compounding public health crises. Shifting temperatures and precipitation patterns could have a profound effect on the future seasonality and spatial spread of SARS-CoV-2 and other viruses either directly or through effects on reservoir species. Moreover, climate change is a major driver of the increased frequency and strength of meteorological disasters, including flooding, hurricanes, and wildfires, leading to, for instance, an increasingly long and severe global wildfire season, which represents a growing source of acute air pollution exposure. Climatic extremes can lead to mass migration, resulting in crowding that can facilitate the spread of infectious diseases, including respiratory infections (30). Extreme climatic events can also severely hamper health infrastructure, making vaccination against and treatment of COVID-19 and other communicable diseases more difficult (30). Future research will be needed to characterize the impact of climate change on the spatiotemporal distribution of viruses and to identify areas where increasing frequency and duration of extreme events compound the risk of disease transmission.

Finally, key uncertainties related to SARS-CoV-2 transmission itself limit a full understanding of the environment–COVID-19 relationship. With the emergence of variants of concern, it remains to be seen how environmental relationships change. Both B.1.1.7 and B.1.351 variants have shown higher receptor binding affinity (109) and could be less sensitive to environmental pressures, though this assumption has not yet been documented. Individuals with the B.1.1.7 variant may be infected for longer (67), potentially leading to more virus in wastewater, in indoor air, and on high-touch surfaces. The severity of B.1.1.7 and B.1.617.2 (delta) variant infections is thought to be increased as compared with previously circulating variants (23, 125), which may lead to compounding mortality risks for individuals who are highly exposed to environmental insults. Age is a critical risk factor for poor outcomes, yet we still do not have fully resolved estimates of age-dependent susceptibility and transmissibility. Similarly, immune age, a combination of immunosenescence and exposure history, may affect susceptibility to COVID-19 infection (123). At the same time, vulnerability to environmental exposures changes over the life course (72, 73), and we do not know how timing of exposures will affect COVID-19 susceptibility and severity.

## CONCLUSIONS

Public health efforts to reduce adverse environmental impacts on COVID-19 incidence and severity would yield substantial public health cobenefits, though much work remains to elucidate the relationships detailed in this review. Global declines in air pollution levels would reduce child mortality from lower respiratory infections; limiting workplace chemical exposures would reduce the risk of cancers; and a restructuring of the built environment could provide more efficient, safe public transit, reduce crowding, expand green space, and promote quality housing. These are highly desirable end points on their own merit, but they would also address risk modifiers for COVID-19 and serve as preventive measures for future pandemics. Adverse environmental exposures are distributed inequitably across geographic, racial, and economic strata, often higher in communities that also lack access to health resources. Special emphasis should be placed on directing assistance and resources to communities with a doubly high burden of environmental pollutants and low access to health, housing, nutrition, and other essential needs.

Even further upstream, the emergence of SARS-CoV-2 has underlined the importance of our relationship with nature. Ongoing population growth, agricultural expansion, habitat destruction, wildlife trade, concentrated animal agriculture, and other major global changes are altering the risk of transmission of zoonotic pathogens (50). Novel pathogens originating from wet markets, wildlife trade, poaching, farming, and suburbanization are consequences of unsustainable human expansion. A rebalancing of our interactions with the environment with the goals of conservation, pollution reduction, and health equity would mark a powerful step toward reducing the impact of—and possibly preventing—future pandemic diseases.

## Supplementary Material

Table S1 - supplementary table
