# Supplementary material for: Environmental Factors Influencing COVID-19 Incidence and Severity
Source: Annu Rev Public Health. Author manuscript; Available in PMC 2023 Mar 28. (PMC10044492; doi:10.1146/annurev-publhealth-052120-101420)
Supplement: Table S1 - supplementary table [file NIHMS1882050-supplement-Table_S1_-_supplementary_table.docx]

**Table S1.** Literature reviewed (including research not selected for discussion in the main text) organized by environmental risk factor and summarized with respect to strength of observed associations and key uncertainties and limitations. Only studies specific to COVID-19 are included.

| **Environmental exposure** | **Specific hazard** | **Literature reviewed** | **Qualitative description of direction and strength of associations** | **Key uncertainties & limitations** |
| --- | --- | --- | --- | --- |
| **Air Pollution** | Particulate matter (including smoke) | Bowe et al. 2021  Coker et al. 2020  Cole et al. 2020  Fattorini and Regoli 2020  Hoffman et al. 2020  Liang et al. 2020  Rohrer et al. 2020  Sagawa et al. 2021  Travaglio et al. 2021  Wu et al. 2020b  Yilin et al. 2015  Zhou et al. 2021  Zhu et al. 2020 | Most ecological studies reviewed found a positive relationship between long-term exposure to particulate matter and increased incidence of and mortality from COVID-19. However, some found non-significant associations. While the number of cohort studies is limited, one reviewed also found a positive relationship.  . | High number of ecological study designs, coarse-scale exposure assessment and misclassification, some residual or uncontrolled confounding, limitations inherent to surveillance data (e.g., disparities in case ascertainment). Remaining uncertainty regarding short-term exposures to high concentrations of pollutants, such as experienced during a wildfire event. |
|  | NO_x_ | Chen et al. 2021  Cole et al. 2020  Fattorini and Regoli 2020  Liang et al. 2020  Lipsitt et al. 2021  Travaglio et al. 2021  Zhu et al. 2020 | Most ecological studies reviewed support a positive association with COVID-19 cases and mortality. While limited, one cohort study reviewed also found a positive association. |  |
|  | Ozone | Fattorini and Regoli 2020  Liang et al. 2020  Travaglio et al. 2021  Zhu et al. 2020 | Limited support for an association between ozone exposure and COVID-19 outcomes. The relatively few studies conducted to date yield mixed results about the significance and directionality of association. |  |

| **Chemicals** | Endocrine disrupting chemicals (e.g., bisphenol A, phthalates) (EDCs) | Wu et al. 2021a | Limited evidence from one computational biology study, which identified several potential pathways as dysregulated by EDC exposure and linked to COVID-19 severity. | A limited number of studies have been conducted to date. There are difficulties isolating the effects of single chemical exposures, the effect of timing throughout the life course, and epigenetic effects. |
| --- | --- | --- | --- | --- |
|  | Metal/Metalloids (e.g., arsenic, cadmium, lead) | Zeng et al. 2021a  Zeng et al. 2021b | Early studies reviewed showed elevated levels of urinary chromium, cadmium, mercury, and lead as well as calcium and chromium in whole blood in patients with severe COVID-19 outcomes. |  |
|  | Per- and Polyfluoroalkyl substances (e.g., PFAS, PFOA) | Grandjean et al. 2020 | Early evidence from one reviewed study that perfluorobutanoic acid (PFBA) in plasma was associated with severe COVID-19. |  |
| **Climate** | Temperature | Biryukov et al. 2020  Carleton et al. 2021  Chin et al. 2020  Dabisch et al. 2021  Liu et al. 2020  Ma et al. 2021  Matson et al. 2020  Merow and Urban 2020  Morris et al. 2021  Nottmeyer and Sera 2021  Raiteux et al. 2021  Riddell et al. 2020  Rubin et al. 2020  Sera et al. 2021  Smith et al. 2021  Wu et al. 2020c | Strong evidence for higher viral stability at lower temperature levels from laboratory studies. However, viral stability may be less sensitive to temperature in the presence of UV light. Mixed epidemiologic results, especially in studies conducted in the earliest phases of the pandemic, as the large susceptible population may minimize the effect of climatic factors. Still, many studies find increased measures of transmission and severity (e.g., R_e_, incidence, mortality rate) at lower temperature levels. Several large, international studies find non-linear associations between temperature and COVID-19 outcomes, with R_e_ peaking between 10°C and 20°C. | High number of ecological study designs, coarse-scale exposure assessment and misclassification, limitations inherent to surveillance data, uncontrolled or residual confounding, limited laboratory studies testing real-world environmental conditions. Outdoor conditions may not reflect indoor conditions, where most transmission occurs. Relatively small signal compared to restrictions on human movement in the short-term. Associations may depend on the proportion of the population immunized, and thus may not be generalizable across time or geographic area. |
|  | Humidity | Biryukov et al. 2020  Carleton et al. 2021  Dabisch et al. 2021  Liu et al. 2020  Ma et al. 2021  Matson et al. 2020  Merow and Urban 2020  Morris et al. 2021  Nottmeyer and Sera 2021  Sera et al. 2021  Smith et al. 2021  Wu et al. 2020c | Strong evidence for higher viral stability at lower humidity levels from laboratory studies, with some non-linear associations observed. Most epidemiologic studies found weak, negative, and non-linear associations between humidity and COVID-19 incidence, R_e_, and mortality rate. |  |
|  | UV radiation | Carleton et al. 2021  Dabisch et al. 2021  Ma et al. 2021  Merow and Urban 2020  Raiteux et al. 2021  Ratnesar-Schumate et al. 2020  Sagripanti and Lytle 2020  Sera et al. 2021  Schuit et al. 2020  Smith et al. 2021 | Strong laboratory evidence for accelerated inactivation of SARS-CoV-2 at higher levels of UV radiation. Mixed results from epidemiologic studies, with some studies finding no association and others finding a negative association between UV radiation and COVID-19 outcomes, including growth rate and R_e_. Difficult to generalize the results of controlled laboratory settings, as most transmission occurs indoors where UV light may be limited. |  |
|  | Population mixing | Damette et al. 2021  Wu et al. 2021d | Limited evidence that daily weather is associated with changes in COVID-19 via changes in contact rate. |  |
| **Built Environment** | Indoor environment (e.g., ventilations/filtration, climate control, sanitation) | Aboubakr et al. 2020  Allen and Ibrahim 2021  Harvey et al. 2021  Lerner et al. 2020  Morawska et al. 2022 | Strong evidence that ventilation and filtration measures reduce concentrations of viral aerosols. Strong support for viral stability under climate-controlled conditions, but limited studies testing stability outside of the laboratory setting. | Limited understanding of the causal effects of the exposome, uncertainty regarding individual-level variation in the exposome and its significance, and misclassification due to the use of neighborhood-level data. |
|  | Physical distancing | Ahmad et al. 2020  Cox-Ganser and Henneberger 2021  Emeruwa et al. 2020  Jay et al. 2020  Leibowitz et al. 2021  Lewnard et al. 2021  Saloner et al. 2020 | Strong evidence that physical distancing reduces SARS-CoV-2 transmission, as well as strong evidence for racial and socioeconomic disparities in ability to physically distance. |  |
|  | Residential segregation and deprivation | Bryan et al. 2020  Das et al. 2021  Hong et al. 2021  Rader et al. 2020  Torrats-Espinosa 2021 | Emerging evidence that residential segregation is associated with poor COVID-19 outcomes. Strong evidence that residential deprivation is associated with poor COVID-19 outcomes. |  |
|  | SARS-CoV-2 exposure disparities | Cox-Ganser and Henneberger 2021  Hong et al. 2021  Jay et al. 2020  Zelner et al. 2021 | Strong evidence that racial minorities and lower-income populations are disproportionately exposed to SARS-CoV-2. |  |

**References cited in supplement**

Aboubakr HA, Sharafeldin TA, Goyal SM. 2020. Stability of SARS‐CoV‐2 and other coronaviruses in the environment and on common touch surfaces and the influence of climatic conditions: A review. *Transbound Emerg Dis*. 68(2):296–312

Ahmad K, Erqou S, Shah N, Nazir U, Morrison AR, et al. 2020. Association of poor housing conditions with COVID-19 incidence and mortality across US counties. *PLoS ONE*. 15(11):e0241327

Allen JG, Ibrahim AM. 2021. Indoor Air Changes and Potential Implications for SARS-CoV-2 Transmission. *JAMA*. 325(20):2112–13

Biryukov J, Boydston JA, Dunning RA, Yeager JJ, Wood S, et al. 2020. Increasing Temperature and Relative Humidity Accelerates Inactivation of SARS-CoV-2 on Surfaces. *mSphere*. 5(4):

Bowe B, Xie Y, Gibson AK, Cai M, van Donkelaar A, et al. 2021. Ambient fine particulate matter air pollution and the risk of hospitalization among COVID-19 positive individuals: Cohort study. *Environment International*. 154:106564

Bryan MS, Sun J, Jagai J, Horton DE, Montgomery A, et al. 2020. COVID-19 mortality and neighborhood characteristics in Chicago. *Annals of Epidemiology*. 56:47–54

Carleton T, Cornetet J, Huybers P, Meng KC, Proctor J. 2021. Global evidence for ultraviolet radiation decreasing COVID-19 growth rates. *Proc Natl Acad Sci USA*. 118(1):e2012370118

Chen Z, Huang BZ, Sidell MA, Chow T, Eckel SP, et al. 2021. Near-roadway air pollution associated with COVID-19 severity and mortality – Multiethnic cohort study in Southern California. *Environment International*. 157:106862

Chin AWH, Chu JTS, Perera MRA, Hui KPY, Yen H-L, et al. 2020. Stability of SARS-CoV-2 in different environmental conditions. *The Lancet Microbe*. 1(1):e10

Coker ES, Cavalli L, Fabrizi E, Guastella G, Lippo E, et al. 2020. The Effects of Air Pollution on COVID-19 Related Mortality in Northern Italy. *Environ Resour Econ (Dordr)*. 76:611–34

Cole MA, Ozgen C, Strobl E. 2020. Air Pollution Exposure and Covid-19 in Dutch Municipalities. *Environ Resour Econ (Dordr)*. 76:581–610

Cox-Ganser JM, Henneberger PK. 2021. Occupations by Proximity and Indoor/Outdoor Work: Relevance to COVID-19 in All Workers and Black/Hispanic Workers. *American Journal of Preventive Medicine*. 60(5):621–28

Dabisch P, Schuit M, Herzog A, Beck K, Wood S, et al. 2021. The influence of temperature, humidity, and simulated sunlight on the infectivity of SARS-CoV-2 in aerosols. *Aerosol Science and Technology*. 55(2):142–53

Damette O, Mathonnat C, Goutte S. 2021. Meteorological factors against COVID-19 and the role of human mobility. *PLoS ONE*. 16(6):e0252405

Das A, Ghosh S, Das K, Basu T, Dutta I, Das M. 2021. Living environment matters: Unravelling the spatial clustering of COVID-19 hotspots in Kolkata megacity, India. *Sustainable Cities and Society*. 65:102577

Emeruwa UN, Ona S, Shaman JL, Turitz A, Wright JD, et al. 2020. Associations Between Built Environment, Neighborhood Socioeconomic Status, and SARS-CoV-2 Infection Among Pregnant Women in New York City. *JAMA*. 324(4):390

Fattorini D, Regoli F. 2020. Role of the chronic air pollution levels in the Covid-19 outbreak risk in Italy. *Environmental Pollution*. 264:114732

Grandjean P, Timmermann CAG, Kruse M, Nielsen F, Vinholt PJ, et al. 2020. Severity of COVID-19 at elevated exposure to perfluorinated alkylates. *PLoS ONE*. 15(12):e0244815

Harvey AP, Fuhrmeister ER, Cantrell ME, Pitol AK, Swarthout JM, et al. 2021. Longitudinal Monitoring of SARS-CoV-2 RNA on High-Touch Surfaces in a Community Setting. *Environ. Sci. Technol. Lett.* 8(2):168–75

Hoffmann M, Kleine-Weber H, Schroeder S, Krüger N, Herrler T, et al. 2020. SARS-CoV-2 Cell Entry Depends on ACE2 and TMPRSS2 and Is Blocked by a Clinically Proven Protease Inhibitor. *Cell*. 181(2):271-280.e8

Hong B, Bonczak BJ, Gupta A, Thorpe LE, Kontokosta CE. 2021. Exposure density and neighborhood disparities in COVID-19 infection risk. *Proc Natl Acad Sci USA*. 118(13):e2021258118

Jay J, Bor J, Nsoesie EO, Lipson SK, Jones DK, et al. 2020. Neighbourhood income and physical distancing during the COVID-19 pandemic in the United States. *Nat Hum Behav*. 4(12):1294–1302

Leibowitz AI, Siedner MJ, Tsai AC, Mohareb AM. 2021. Association Between Prison Crowding and COVID-19 Incidence Rates in Massachusetts Prisons, April 2020-January 2021. *JAMA Intern Med*. 181(10):1315

Lerner AM, Folkers GK, Fauci AS. 2020. Preventing the Spread of SARS-CoV-2 With Masks and Other “Low-tech” Interventions. *JAMA*. 324(19):1935

Lewnard JA, Mora AM, Nkwocha O, Kogut K, Rauch SA, et al. 2021. Prevalence and Clinical Profile of Severe Acute Respiratory Syndrome Coronavirus 2 Infection among Farmworkers, California, USA, June–November 2020. *Emerg. Infect. Dis.* 27(5):1330–42

Liang D, Shi L, Zhao J, Liu P, Sarnat JA, et al. 2020. Urban Air Pollution May Enhance COVID-19 Case-Fatality and Mortality Rates in the United States. *The Innovation*. 1(3):100047

Lipsitt J, Chan-Golston AM, Liu J, Su J, Zhu Y, Jerrett M. 2021. Spatial analysis of COVID-19 and traffic-related air pollution in Los Angeles. *Environment International*. 153:106531

Liu J, Zhou J, Yao J, Zhang X, Li L, et al. 2020. Impact of meteorological factors on the COVID-19 transmission: A multi-city study in China. *Sci Total Environ*. 726:138513

Ma Y, Pei S, Shaman J, Dubrow R, Chen K. 2021. Role of meteorological factors in the transmission of SARS-CoV-2 in the United States. *Nat Commun*. 12(1):3602

Merow C, Urban MC. 2020. Seasonality and uncertainty in global COVID-19 growth rates. *Proc Natl Acad Sci USA*. 117(44):27456–64

Morawska L, Allen J, Bahnfleth W, Bluyssen PM, Boerstra A, et al. 2021. A paradigm shift to combat indoor respiratory infection. *Science*. 372(6543):689–91

Morris DH, Yinda KC, Gamble A, Rossine FW, Huang Q, et al. 2020. The effect of temperature and humidity on the stability of SARS-CoV-2 and other enveloped viruses. . bioRxiv 341883

Nottmeyer LN, Sera F. 2021. Influence of temperature, and of relative and absolute humidity on COVID-19 incidence in England - A multi-city time-series study. *Environ Res*. 196:110977

Rader B, Astley CM, Sy KTL, Sewalk K, Hswen Y, et al. 2020. Geographic access to United States SARS-CoV-2 testing sites highlights healthcare disparities and may bias transmission estimates. *Journal of Travel Medicine*. 27(7):

Raiteux J, Eschlimann M, Marangon A, Rogée S, Dadvisard M, et al. 2021. Inactivation of SARS-CoV-2 by Simulated Sunlight on Contaminated Surfaces. *Microbiol Spectr*. 9(1):

Ratnesar-Shumate S, Williams G, Green B, Krause M, Holland B, et al. 2020. Simulated Sunlight Rapidly Inactivates SARS-CoV-2 on Surfaces. *The Journal of Infectious Diseases*. 222(2):214–22

Riddell S, Goldie S, Hill A, Eagles D, Drew TW. 2020. The effect of temperature on persistence of SARS-CoV-2 on common surfaces. *Virol J*. 17(1):145

Rohrer M, Flahault A, Stoffel M. 2020. Peaks of Fine Particulate Matter May Modulate the Spreading and Virulence of COVID-19. *Earth Syst Environ*. 4(4):789–96

Rubin D, Huang J, Fisher BT, Gasparrini A, Tam V, et al. 2020. Association of Social Distancing, Population Density, and Temperature With the Instantaneous Reproduction Number of SARS-CoV-2 in Counties Across the United States. *JAMA Netw Open*. 3(7):e2016099

Sagawa T, Tsujikawa T, Honda A, Miyasaka N, Tanaka M, et al. 2021. Exposure to particulate matter upregulates ACE2 and TMPRSS2 expression in the murine lung. *Environmental Research*. 195:110722

Sagripanti J, Lytle CD. 2020. Estimated Inactivation of Coronaviruses by Solar Radiation With Special Reference to COVID‐19. *Photochem Photobiol*. 96(4):731–37

Saloner B, Parish K, Ward JA, DiLaura G, Dolovich S. 2020. COVID-19 Cases and Deaths in Federal and State Prisons. *JAMA*. 324(6):602

Schuit M, Ratnesar-Shumate S, Yolitz J, Williams G, Weaver W, et al. 2020. Airborne SARS-CoV-2 Is Rapidly Inactivated by Simulated Sunlight. *The Journal of Infectious Diseases*. 222(4):564–71

Sera F, Armstrong B, Abbott S, Meakin S, O’Reilly K, et al. 2021. A cross-sectional analysis of meteorological factors and SARS-CoV-2 transmission in 409 cities across 26 countries. *Nature Communications*. 12(1):5968

Smith TP, Flaxman S, Gallinat AS, Kinosian SP, Stemkovski M, et al. 2021. Temperature and population density influence SARS-CoV-2 transmission in the absence of nonpharmaceutical interventions. *Proc Natl Acad Sci USA*. 118(25):e2019284118

Torrats-Espinosa G. 2021. Using machine learning to estimate the effect of racial segregation on COVID-19 mortality in the United States. *Proc Natl Acad Sci USA*. 118(7):e2015577118

Travaglio M, Yu Y, Popovic R, Selley L, Leal NS, Martins LM. 2021. Links between air pollution and COVID-19 in England. *Environmental Pollution*. 268:115859

Wu Q, Coumoul X, Grandjean P, Barouki R, Audouze K. 2021. Endocrine disrupting chemicals and COVID-19 relationships: A computational systems biology approach. *Environment International*. 157:106232

Wu X, Nethery RC, Sabath MB, Braun D, Dominici F. 2020. Air pollution and COVID-19 mortality in the United States: Strengths and limitations of an ecological regression analysis. *Sci. Adv.* 6(45):eabd4049

Wu Y, Jing W, Liu J, Ma Q, Yuan J, et al. 2020. Effects of temperature and humidity on the daily new cases and new deaths of COVID-19 in 166 countries. *Science of The Total Environment*. 729:139051

Wu Y, Mooring TA, Linz M. 2021. Policy and weather influences on mobility during the early US COVID-19 pandemic. *Proc Natl Acad Sci USA*. 118(22):e2018185118

Yilin Z, Yandong N, Faguang J. 2015. Role of angiotensin-converting enzyme (ACE) and ACE2 in a rat model of smoke inhalation induced acute respiratory distress syndrome. *Burns*. 41(7):1468–77

Zelner J, Trangucci R, Naraharisetti R, Cao A, Malosh R, et al. 2021. Racial Disparities in Coronavirus Disease 2019 (COVID-19) Mortality Are Driven by Unequal Infection Risks. *Clinical Infectious Diseases*. 72(5):e88–95

Zeng H-L, Yang Q, Yuan P, Wang X, Cheng L. 2021. Associations of essential and toxic metals/metalloids in whole blood with both disease severity and mortality in patients with COVID-19. *The FASEB Journal*. 35(3):e21392

Zeng H-L, Zhang B, Wang X, Yang Q, Cheng L. 2021. Urinary trace elements in association with disease severity and outcome in patients with COVID-19. *Environmental Research*. 194:110670

Zhou X, Josey K, Kamareddine L, Caine MC, Liu T, et al. 2021. Excess of COVID-19 cases and deaths due to fine particulate matter exposure during the 2020 wildfires in the United States. *Sci. Adv.* 7(33):eabi8789

Zhu Y, Xie J, Huang F, Cao L. 2020. Association between short-term exposure to air pollution and COVID-19 infection: Evidence from China. *Science of The Total Environment*. 727:138704
